# A pilot single-blind parallel randomised controlled trial comparing kinesiology tape to compression in the management of subacute hand oedema after trauma

**DOI:** 10.1186/s40814-022-01023-1

**Published:** 2022-03-26

**Authors:** Leanne Miller, Christina Jerosch-Herold, Lee Shepstone

**Affiliations:** 1 Therapies Department, Outpatients East Level 2, Norfolk and Norwich University Hospital, Colney Lane, Norwich, NR4 7UY UK; 2 School of Health Sciences, Queen’s Building, University of East Anglia, Norwich Research Park, Norwich, NR4 7TJ UK; 3 Norwich Medical School, University of East Anglia, Norwich Research Park, Norwich, NR4 7TJ UK

**Keywords:** Hand, Oedema, Compression, Kinesiology tape, Pilot, Randomisation, Trial, Feasibility, Adherence

## Abstract

**Background:**

Hand oedema is a common consequence of hand trauma or surgery. There are numerous methods to reduce hand oedema but lack high-quality evidence to support best practice. The primary objective of this pilot trial was to assess study feasibility when comparing treatments for subacute hand oedema after trauma.

**Methods:**

A parallel two-arm pilot randomised controlled trial was conducted in the hand therapy department at a regional hospital in Norfolk between October 2017 and July 2018. Patients were eligible if 18 years or over, referred to hand therapy with subacute hand oedema. Randomisation was on a 1:1 basis to treatment as usual (TAU) (compression, elevation and massage) or trial treatment (TT) (kinesiology tape, elevation and massage). One blinded assessor completed all assessments (prior to randomisation, 4 and 12 weeks later). Data on study feasibility, adherence and acceptability of treatments were collected. The primary outcome measure was hand volume (volumetry). Patient-rated severity (0–5 Likert scale), hand health profile of the Patient Evaluation Measure (PEM) and quality of life (EQ-5D-5L) were also recorded.

**Results:**

Forty-five patients were screened for eligibility and 26 consented and were randomised with 13 patients in each treatment arm. Twelve participants were lost to follow-up leaving 7 participants in each group included in the analysis. Assessor blinding was maintained in 64% of participants (9/14). Total mean acceptability scores, out of 100, were higher for TAU (87.9) than TT (76.1). Health resource use results showed TT was marginally cheaper (~£2 per patient) than TAU.

Individual adherence ranged between 39 and 100%, with higher levels of overall adherence seen in the TAU group. Four participants (28%) reported adverse effects (TT group *n* = 3, TAU group *n* = 1).

**Conclusion:**

This pilot trial has identified that modifications are required in order to make a full-scale trial feasible. They include a formal assessment of treatment fidelity, research staff assisting with screening and recruitment of participants and multiple blinded assessors at each study site. Whilst not designed as an efficacy trial, it should be acknowledged that the small sample size and high loss to follow-up meant very small numbers were included in the final analysis resulting in wide confidence intervals and therefore low precision in parameter estimates.

**Trial registration:**

International Standard Randomised Controlled Trial Number: 94083271. Date of registration 16th August 2017.

**Trial funding:**

National Institute for Health Research Trainees Co-ordinating Centre (TCC); Grant Codes: CDRF-2014-05-064

**Supplementary Information:**

The online version contains supplementary material available at 10.1186/s40814-022-01023-1.

## Key messages regarding feasibility

1. What uncertainties existed regarding the feasibility?
  The capacity of the study team to screen, consent, recruit and assess enough participants
  The retention of study participants for the 12-week trial period
  The acceptance of the treatments by study participants and use of a paper adherence diary
2. What are the key feasibility findings?
  A formal assessment of skill acquisition and treatment fidelity would be beneficial.
  Strategies to improve retention, such as reminders messages and assessments in patients’ home, should be considered
  The use of multiple blinded assessors and research nurses to assist in screening patients may increase recruitment numbers.
3. What are the implications of the feasibility findings for the design of the main study?
  A multicentre approach is needed to reach the target sample size, along with a local PI in each site, not involved in the recruitment or treatment delivery to oversee study protocols.
  A more in-depth and detailed provider training plan, competency self-assessment before and after teaching and a formal assessment of treatment fidelity (treatment delivery, receipt and enactment) should be included in the trial protocol
  Greater emphasis on educating patients regarding the need to return for follow-up, even if their symptoms have resolved along with follow-up assessment reminders to reduce non-attendance rates is required.

## Background

Oedema is an abnormally large accumulation of interstitial fluid [1] which collects at the site of an injury during the healing phase and can be slow to dissipate. To maximise restitution of the hand following an injury, it is paramount to control oedema effectively [2]. An oedematous hand loses flexibility, strength and precision with dexterous tasks, as the increased fluid can compress peripheral nerves, which act as the hand’s sensory and motor communication channels. Watson-Jones [3] described oedema as “glue” which highlights that prolonged oedema can cause fixed joint contractures, leading to loss of function and long-term disability.

There is no data on the incidence or prevalence of hand oedema as it is a sequalae of hand trauma or surgery for elective or traumatic hand conditions. An internal audit of hand therapy notes identified 90% of patients required treatment for hand oedema. The treatment of oedema forms a core component of a hand therapist’s management of patients with hand conditions.

Many different interventions are used to reduce hand oedema. Sixteen oedema management interventions were identified in the literature [4]. These include traditional methods such as compression (i.e. glove, finger sleeve or compressive wrap), elevation and massage, which are often seen as the mainstays of oedema management, but also newer methods such as kinesiology tape (an adherent elasticated tape). Compression, elevation and massage were identified as the most commonly used treatments for subacute hand oedema in a survey of UK members of the British Association of Hand Therapists (BAHT) in 2015/2016 [5]. For this reason, they were used as the control treatment in this pilot trial.

Kinesiology tape is designed to mimic the elastic properties of the skin by lifting the skin to allow greater interstitial space and encourage lymphatic drainage. As the tape is elastic and stretches up to 55–60% of its length, it allows for unrestricted movement [6, 7]. It leaves the volar surface of the hand free to allow sensory feedback, which is essential for functional use. The tape can also be worn in water.

Whilst these different methods may appear to be effective in a clinical setting, there is little empirical evidence to support their use, and clinicians often employ a range of methods through trial and error. A systematic review [4] found limited low to moderate quality evidence to support the use of a combination of interventions (in addition to standard care), known as manual oedema mobilisation or modified manual lymph drainage, when treating problematic subacute hand oedema compared to standard treatment alone. The results need to be interpreted with caution however due to numerous limitations associated with the included studies. The various methods employed to treat oedema, which are often prescribed in conjunction with each other, have different proposed modes of action. Elevation uses gravitational forces to enhance the flow of oedema away from the limb [8], massage relies upon the stimulation of lymphatic system and mobilisation of the fluid [9], and compression acts as an external counter pressure [10, 11] which pushes the fluid proximally in to the venous and lymphatic systems [12, 13]. In contrast, kinesiology tape proposedly creates a pulling force on the skin which allows greater interstitial space and encourages lymphatic drainage [6].

There is a lack of scientific corroboration of these proposed mechanisms of action; therefore, comparing treatments in a clinical trial, when the treatments themselves are not fully understood, creates further uncertainties. For these reasons, oedema management could be viewed as a complex intervention [14] where it is acknowledged that the evaluation of these “is difficult because of problems developing, identifying, documenting, and reproducing the intervention”. No previous studies have compared kinesiology tape with compression, which propose opposing mechanisms of action, in patients with subacute hand oedema. A definitive (phase III) randomised controlled trial is premature. There is a need to collect preliminary information, to inform a definitive trial and to ensure that all the components of the study run as proposed.

## Methods

### Study design and setting

A pilot, single-blind, parallel, randomised controlled trial was conducted between 30th October 2017 and 31st July 2018 in the hand therapy department of the Norfolk and Norwich University Hospital. This is a regional specialist hospital for orthopaedics and plastic surgery and covers a catchment area of approximately 1,016 000 people [15].

The focus of the study was on the processes of recruitment, randomisation, treatment and follow-up assessments. An assessment of the collection of the primary outcome was made, and a comparison was made between the study groups as a preliminary estimate of treatment efficacy.

Ethical approval to conduct the study was obtained by the East of Scotland Research Ethics Service (REC ref: 17/ES/0098), the Health Research Authority (HRA) and Research Governance Department at the Norfolk and Norwich Hospital.

### Eligibility and recruitment

Eligible participants were aged 18 years and over, referred to the outpatient hand therapy department after trauma or surgery, able to give informed consent, and for whom treatment of sub-acute hand oedema was indicated, as confirmed by their treating therapist. Subacute was defined as oedema which presented from 3 days up to 6 weeks after trauma or surgery. Due to low recruitment numbers and a re-evaluation of the literature this timeframe was amended during the recruitment phase to include oedema present up to 12 weeks following trauma or surgery. This amendment received HRA approval. Patients were excluded if their oedema was longer than 12 weeks in duration or if they had already commenced oedema management treatments. Other exclusions were as follows: tendon repairs within the first 4 weeks, patients with chronic vascular, cardiac or lymphatic conditions or any other physical or mental health condition which would have affected the patient’s ability to safely apply and monitor the use of compression or kinesiology tape. Complex regional pain syndrome (CRPS) was not an exclusion criterion, and it is plausible that participants may have developed CRPS whilst enrolled in the trial.

Participants were screened for eligibility by their treating hand therapist who introduced the study to them. Patients who met the eligibility criteria and provided verbal consent to take part were formally recruited by the principal investigator (PI) who explained the study in detail and obtained written consent.

### Allocation and randomisation

Participants were randomly allocated on a 1:1 basis to either the intervention arm/trial treatment (TT) which consisted of kinesiology tape, elevation and massage or the control arm, treatment as usual (TAU) including compression, elevation and massage. The trial statistician generated the block randomised allocation sequence (block length of 2, 4 or 6). A therapy assistant, who was not involved with the trial, prepared sequentially numbered opaque sealed envelopes which housed the intervention allocation. These envelopes were stored in a lockable storeroom in the hand therapy room.

### Interventions

Table 1 provides a description of the interventions following the TIDieR (template for the intervention description and reporting) structure recommended by the Equator network [16] in conjunction with the CONSORT statement [17].

**Table 1:** TiDier table describing control and intervention group treatments

Name	Treatment as usual (TAU)	Trial treatment (TT)
**Why**	*Compression* for hand oedema is usually achieved through Lycra gloves which exerts around 35 +/− 5 mmHg pressure on the tissues of the hand [10]. The garment acts as an external counter pressure [10, 11] which compensates for the inelasticity of oedematous tissues and therefore improves circulatory efficiency by facilitating venous and lymphatic flow [11, 13] *Massage* techniques are used to stimulate the lymphatic system [13]. Different methods are documented in the literature which employ various degrees of force or pressure on the skin directing the oedema towards regional lymph nodes. Traditional ‘retrograde massage’ uses a moderate force ‘milking’ action but is considered too aggressive for the delicate lymphatic system to cope with and has been questioned [8]. Instead, a lighter tractioning of the skin has been proposed in a longitudinal direction to produce a stretch reflex to the skin [11]. Both methods are used in clinical practice *Elevation* permits gravity to assist with the drainage of oedema from the distal limb [8]. Elevation alone [3] is not effective in reducing oedema but is recommended in combination with other modalities	***Kinesiology tape*** is designed to mimic the elastic properties of the skin by lifting the skin to allow greater interstitial space and encourage lymphatic drainage. In contrast to the traditional compression method, it is designed to push the fluid proximally into the venous and lymphatic system [6]. The tape is said to be unique in that it mimics the elastic properties of the skin and its wave-like grain provides a pulling force to the skin creating more space by lifting the fascia and soft tissues under the areas where it is applied [18]. This multifunctional tape can be applied anywhere on the face or body. The benefit of using it in the hand, unlike an oedema glove or other form of compression, is that it leaves the majority of the skin surface free for sensory feedback which is essential for functional use. It can also be worn in water. As the tape is elastic and stretches up to 55–60% of its length, it also allows for unrestricted movement [18, 19] **Massage:** as per TAU **Elevation:** as per TAU
**What — materials**		
	
**What — procedures**	Standardised oedema management programmes designed through an Internet-mediated Delphi consensus method with 8 volunteer hand therapy experts. The standardised programme was then made into a patient instruction leaflet which was made accessible to patients during a process of meetings and reviews with a patient advisory committee	
**Who**	Treatment was demonstrated to patients by members of the hand therapy team. These are occupational or physical therapists who specialise in hand therapy Hand therapists regularly advise patients about managing their oedema following injury or surgery and prescribe a combination of compression, elevation and massage as required All therapists involved in the trial were trained by the PI on the treatment protocol and method of implementing each treatment	
**How**	All therapy sessions were delivered on a face-to-face individual basis. The therapist equipped the participant with the materials required to self-administer the programme unsupervised at home. This included the application of kinesiology tape and compression as advised by their therapist and supported by a written information sheet	
**When and how much**	Wear for 20–24 h a day, removing for hygiene for up to 12 weeks	Applied to the skin full time for 3–5 days. No tension at the proximal anchor, 0–25% tension of the central tape
	**Massage:** 5–10 min, 3–6 times a day for at least 2 weeks or until the swelling has resolved **Elevation:** As much as possible during the day and night when the hand is not being used. Continued until the patient and therapist mutually agree the oedema has subsided	
**Tailoring**	Latex-free versions available	A 24-h rest period can be utilised between application but is not essential if there has been no issues
	**Massage:** Reduce frequency and duration if unable to tolerate massage or if a smaller area is affected **Elevation:** Active elevation or using Bradford sling in the day and Bradford sling or pillow day or night	
**Modifications**	Remove if vascularity compromised	Remove in cases of skin irritation
	**Massage:** Discontinue if pain or swelling increases **Elevation:** Discontinue if pain (in neck, shoulder or elbow), sensation or symptoms worsen or if vascularity compromised (colour changes to digits)	
**How well**	There were no planned or actual assessments of treatment fidelity. A patient adherence diary was used to record the extent to which treatments were adhered to on a weekly basis, either not at all, in part of as advised	

### Outcomes

#### Hand volume (Fig. 1)

The primary outcome measure was a single volume measure of the affected hand in millimeters using a volumeter (water displacement method). This has been referred to as the “gold standard” method of measuring hand volume, having excellent inter and intra-rater reliability [20] and responsiveness [21] and was the most responsive outcome measure from an observational study of 73 participants with handoedema (unpublished).

**Fig. 1:**
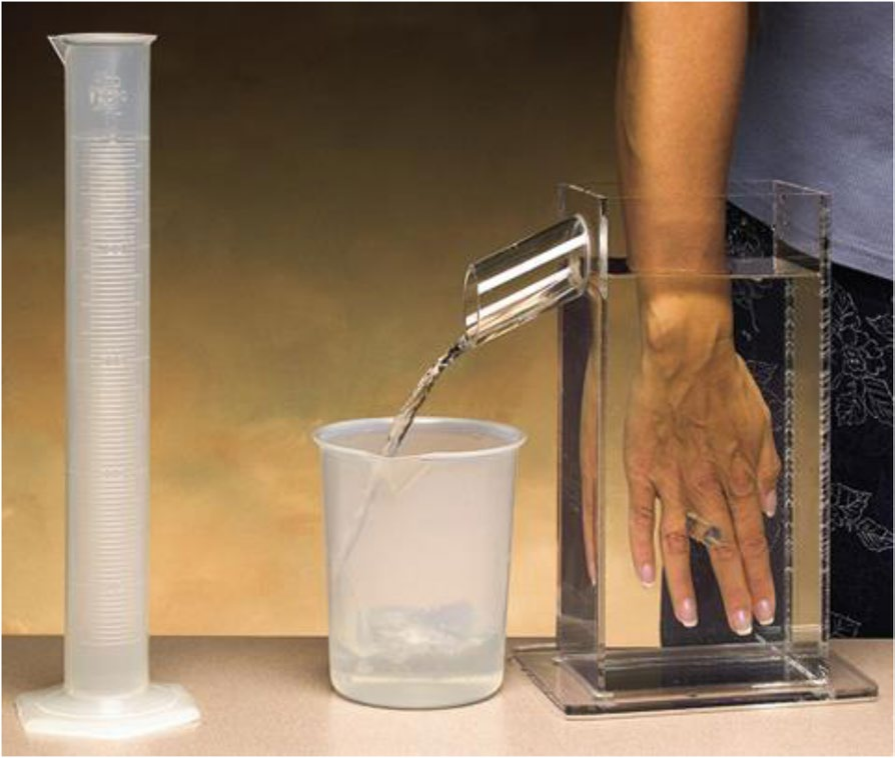
The volumeter set

#### Patient-rated oedema severity

The Oedema Rating Scale (ORS) is a self-reported severity-of-swelling scale where the participant is asked to “rate the swelling in your hand today”, using a 7-point ordinal scale (0 = none, 1 = minimal, 2 = mild, 3 = moderate, 4 = severe, 5 = very severe, 6 = extreme). The ORS had been devised in collaboration with a patient advisory group made up of current and previous hand therapy patients and was used in this study to record perceived change in oedema.

#### Patient-rated functional scale

The hand health profile of the patient evaluation measure (PEM) [22] is a validated 11-item region-specific, patient-reported outcome measure (PROM) which was used to record changes in functional ability in this pilot trial. It is scored on a 1–7 Likert scale, with the total combined score being expressed as a percentage: the higher the score, the greater the perceived disability. Unlike other commonly used region-specific PROMS, the PEM includes items on “feel” and “appearance” of the hand, which may relate to swelling. The PEM is a reliable, valid and responsive instrument in assessing outcomes of disorders of the hand [22].

#### Patient-rated quality of life

The EQ-5D-5L [23] is a standardised measure of health status which aims to provide “a simple generic measure of health for clinical and economic appraisal” [24]. It has been recommended by the Chartered Society of Physiotherapists (CSP) to be used to measure change in musculoskeletal outpatient settings [25]. The EQ-5D-5L has two parts to it, the first asks 5 questions relating to mobility, self-care, usual activities, pain/discomfort and anxiety/depression with 5 levels of responses from no problems to not being able to complete the task or having extreme symptoms- the sum of which is referred to as the utility score. The second part is a 0–100 visual analogue scale (VAS) where participants are asked to rate their health today.

#### Acceptability of oedema treatment

A brief questionnaire was designed (see Additional file 1) to assess how acceptable participants’ found the oedema treatments which the PI completed with all participants after their final follow-up assessment (week 12). The questionnaire consisted of 10 factors relating to the acceptability of the oedema treatment provided, such as ease of use, durability and aesthetics, which the patient was asked to grade on a scale of zero (negative) to 10 (positive). The final open question requested feedback from patients on any aspect not covered by the questionnaire with responses being recorded verbatim.

### Adherence

A simple paper diary was designed (see Additional file 2), based on best practice recommendations [26]. Apart from asking patient to indicate their treatment allocation, the diaries were anonymous. They were placed in a box at reception or handed to their treating therapist or the PI after their final assessment so as not to unblind the assessor. The diary asked participants to identify, for each element of their treatment (i.e. massage, elevation, kinesiology tape/compression) and for each of the 12 weeks of the study, if they had completed their allocated treatment “not at all”, “in part” or “as advised”.

### Health resource use

The number of visits to hand therapy, consumable usage, grade of treating hand therapist and total time spent treating oedema (in therapy appointments) were also recorded to obtain preliminary data on healthcare use and cost.

### Blinding

One experienced hand therapist (study PI) who was blinded to treatment allocation assessed all participants at baseline. The blinded assessor would leave the room after taking consent from the patient during which time the treating hand therapist would randomise the participant and inform the participant which treatment arm they had been allocated to. Participants were reassessed at 4 and 12 weeks by the same blinded assessor.

### Sample size

As a pilot study, principally conducted to assess the suitability of the chosen research methods, the sample size was not based upon the principles of statistical precision nor statistical power for hypothesis testing. Instead, we aimed to recruit 100 patients in a 6-month period which we believed to be practical based on the throughput of patients being referred to the department and on a preliminary review of 10 randomly selected sets of patients’ hand therapy notes which identified 90% of patients required some form of oedema management. We anticipated a loss to follow-up of 20–30% (which was based on the attrition rate of a previous observational study investigating hand oedema), thus providing 70 to 80 completing participants.

### Statistical analysis

Mean and standard deviations (SD) were calculated for each outcome at each time point, along with the mean change from baseline to 4 and baseline to 12 weeks. The level of missing data was assessed and compared with baseline characteristics to identify which groups of participants, if any, were likely to less likely to return full data. A general linear model was used to estimate the effect of kinesiology tape relative to the control, with respect to the effectiveness outcomes. This included the baseline value as a covariate and treatment arm as a fixed effect. Results for the ORS at 4 and 12 weeks were dichotomised into those participants scoring 0–2 (none, minimal, mild) and those scoring 3–6 (moderate, severe, very severe, extreme) before a logistic regression model was constructed. The between-group differences were estimated with 95% confidence intervals though as a pilot study, it was not intended for any conclusion regarding effectiveness to be reached, or indeed, should be reached. The analysis was based on the intention-to-treat principle (analysed by group allocated to); however, there were no plans for imputation of missing data. No subgroup analyses were performed.

Descriptive statistics were used to describe patient flow, particularly estimating the proportion of eligible patients consenting to take part, the frequency of precluding eligibility criteria, and the frequency of losses to follow-up, including active withdrawals (with the reason, where available). Each proportion was calculated with a 95% confidence interval.

Patient-reported adherence was calculated for each participant as a proportion of the total adherence over 12 weeks (treatment which was the frequency and duration “as advised” for all 3 elements of their allocated treatment according to the standardised protocol and patient instruction booklet). This was summarised as a mean with 95% confidence interval.

All analyses were completed using the Statistical Package for Social Sciences (SPSS), version 25.

## Results

**Fig. 2:**
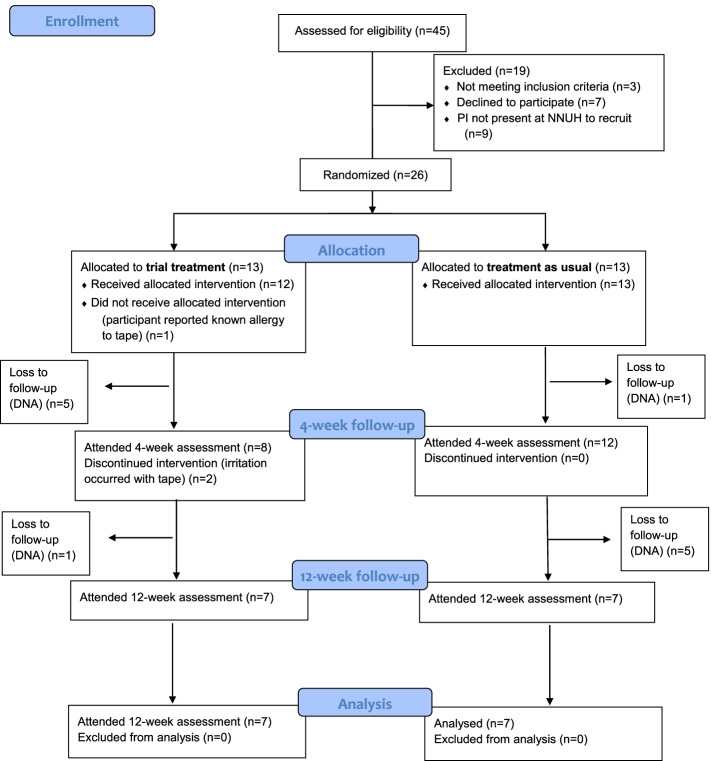
CONSORT flow diagram

Forty-five patients were assessed for eligibility and 26 consented and were randomised. Figure 2 shows the CONSORT diagram [27]. Baseline characteristics of both groups are shown in Table 2.

**Table 2:** Baseline characteristics table

	Treatment as usual (***N*** = 7)	Trial treatment (***N*** = 7)
Gender male:female	4:3	2:5
Age—mean (SD)	63.6 (19.3)	60.0 (17.6)
Affected hand—left: right	3:4	4:3
Location of oedema isolated digit: global	2:5	3:4
Reason for oedema—trauma: surgery	4:3	4:3
Days since injury—mean (range)	39.3 (2159)	27.3 (3–45)
Past medical history–	OA- not hand specific (*n* = 2)	OA — not hand specific (*n* = 3)
	Neuralgia	Type I DM
	Type II DM	Type II DM
	HTN (*n* = 2)	HTN
	COPD	SOB
	Deaf	Under active thyroid
		Anxiety
Condition or operation		
Distal radius fracture (conservative)	*n* = 1 (14%)	*n* = 2 (28%)
Dupuytren’s release	*n* = 1 (14%)	*n* = 1 (14%)
Fracture/dislocation (digit)	*n* = 2 (28%)	*n*= 1 (14%)
Tendon repair and DR fracture	*n* =1 (14%)	*n* = 0
Distal radius fracture fixation	*n* = 1 (14%)	*n* = 2 (28%)
Fracture/dislocation metacarpal	*n* = 1 (14%)	*n* = 0
Joint replacement	*n* = 0	*n* = 1 (14%)

*OA* Osteoarthritis, *HTN* Hypertension, *COPD* Chronic obstructive pulmonary disease, *SOB* Shortness of breath, type II *DM* Diabetes mellitus

Baseline characteristics were similar, with the exception of “time since injury” which was 12 days longer in the treatment as usual group, indicating more chronic oedema.

### Health resource use

Taking into account therapy staff costs and consumable use, the trial treatment was £2.31 cheaper per participant and required on average one fewer visit to hand therapy during the trial period than those in the treatment as usual group. It is likely that the health resource use costs were underestimated due to treating therapists forgetting to document consumable use.

### Adverse effects

Two participants (14%), allocated to the trial treatment group, experienced adverse effects issues with kinesiology tape which resulted in them switching to treatment as usual. These participants reported a rash with small bumps under the skin which were sore to touch, like blisters or that the tape pulled their skin. One other patient in the trial treatment group also reported an itchy rash at her elbow crease where the tape starts; she took a 24-h rest period from the tape as is advised and continued without any further issues. One participant in the treatment as usual group reported adverse effects issues with bruising to the hand which he stated to be as a result of using the compression glove for 24 h. Following reassessment by a hand therapist, he continued with treatment as usual, although it was not used “as advised” for the first 6 days because of this.

### Acceptability

Total mean acceptability score was 76.1 out of 100 for TT and 89.7 for TAU. The largest differences in mean scores were for overall acceptability of treatment and comfort where treatment as usual scored 2.4 and 2.3 points, respectively, more than trial treatment. Comments received from participants regarding the acceptability of treatments are shown in Additional file 3.

### Adherence

Thirteen (93%) adherence diaries were returned. Results show consistently higher levels of adherence across the three elements of TAU than TT over the 12-week trial period. The treatment most adhered to in the TAU group (*n* = 6 diaries) was massage (mean adherence 89.9%, 95% *CI* 74.2–100), followed by compression (83.8%, 95% *CI* 63.7–100), then elevation (57.9%, 95% *CI* 10.4–100). In the trial treatment group (*n* = 7 diaries), kinesiology tape had the highest adherence rate (mean adherence 63.7%, 95% *CI* 37.5–89.9, *n* = 7 diaries), followed by elevation (53.7%, 95% *CI* 15.6–91.8) then massage (48.2%, 95% *CI* 16.7–79.7). Individual adherence ranged from 39 to 100%. Table 3 shows cumulative adherence rates.

**Table 3:** Cumulative adherence rates

Participant	Treatment as usual						Overall	Participant	Trial treatment						Overall
	Massage		Elevation		Compression				Massage		Elevation		Elasticated tape		
**1**	77.8%	7/9	88.9%	8/9	100%	9/9	**88.8%**	**1**^**a**^	50%	6/12	50%	6/12	100%	12/12	**66.6%**
							**24/27**								**24/36**
**2**	100%	4/4	100%	4/4	100%	4/4	**100%**	**2**^**a**^	25%	3/12	100%	12/12	16.6%	2/12	**47.2%**
							**12/12**								**17/36**
**3**	66.6%	8/12	41.6%	5/12	100%	12/12	**69.4%**	**3**	100%	5/5	0%	0/5	60%	3/5	**53.3%**
							**25/36**								**8/15**
**4**	91.6%	11/12	0%	0/12	58.3%	7/12	**50%**	**4**	16.7%	1/6	33.3%	2/6	66.7%	4/6	**38.8%**
							**18/36**								**7/18**
**5**	100%	12/12	16.6%	2/12	66.6%	8/12	**61.1%**	**5**	41.6%	5/12	83.3%	10/12	91.6%	11/12	**72.2%**
							**22/36**								**26/36**
**6**	100%	9/9	100%	9/9	77.8%	7/9	**92.6%**	**6**	55.6%	5/9	55.6%	5/9	44.4%	4/9	**51.9%**
							**25/27**								**14/27**
**7**	Did not return diary							**7**	Not completed		Not completed		66.7%	6/9	**66.7%**
															**6/9**
**Mean**	**89.3%**		**57.9%**		**83.8%**			**Mean**	**48.2%**		**53.7%**		**63.7%**		
**95% CI**	**74.2–100**		**10.4–100**		**63.7–100**			**95% CI**	**16.7–79.7**		**15.6–91.8**		**37.5–89.9**		

Cumulative adherence as a proportion (weeks) and percentages based on the frequency and duration as advised, summarised as a mean adherence for each treatment modality and associated 95% confidence interval for actual treatment time, where known (*n*=7)

^a^Indicates participants who switched treatment from the tape to the glove

### Assessor blinding

After completing the 12 weeks assessment, the blinded assessor was asked to guess which treatment group participants had been allocated to, if blinding had been maintained up to this point. Assessor blinding was maintained in 9 of the 14 participants (64%) throughout the study period. Of these 9, the assessor guessed the correct allocation on 6 occasions (66.6%). Blinding was not maintained in five cases for a variety of reasons which included the assessor seeing the patient with their compression glove in situ, a participant asking the assessor for a new compression glove and a therapist discussing a participant’s allocated treatment with the assessor.

### Treatment effectiveness

A greater mean change was seen in the TT group for hand volume, PEM and EQ-5D-5L VAS scores. Mean change for ORS favoured the TAU group, whereas EQ-5D-5L utility scores were similar, but slightly in favour of the trial treatment. There were no statistically significant differences between TAU and TT in any of the objective or patient-rated outcome measures at 4 weeks or 12 weeks. Results indicate that the participants in the TAU group had 1.6 (baseline to 4 weeks) and 4 times (baseline to 12 weeks) the odds of having the better ORS scores than the TT group. EQ-5D-5L utility scores showed no change since the 4-week assessment. EQ-5D-5L visual analogue scale (VAS) scores improved slightly in the TAU group but remained similar in the TT group. UK population norms for the EQ-5D-5L do not exist. Table 4 gives the mean and standard deviations for all outcome measures at baseline, 4 and 12 weeks. Tables 5 and 6 shows the intention to treat (ITT) analysis for all outcomes as 4 weeks and 12 weeks, respectively.

**Table 4:** Outcomes at baseline, 4 and 12 weeks — mean (standard deviation)

	TAU Baseline mean (SD)	TAU 4-week mean (SD)	TAU 12-week mean	TAU mean change baseline—12 weeks (SD)	TT Baseline mean (SD)	TT 4-week mean (SD)	TT 12-week mean (SD)	TT Mean change baseline—12 weeks (SD)
**Volumeter (ml)**	507.86 (70.23)	490.71 (59.47)	473.57 (60.60)	34.29 (27.75)	505.00 (102.27)	476.43 (103.27)	460.00 (97.47)	45.00 (48.22)
**PEM (0–100)**	54.17 (16.98)	46.20 (19.39)	38.60 (18.15)	15.57 (18.18)	62.70 (15.61)	44.90 (14.57)	36.31 (16.98)	26.39 (16.40)
**ORS (0–6)**	3.14 (0.69)	2.43 (0.79)	1.57 (0.79)	1.57 (0.98)	3.57 (0.79)	2.57 (1.13)	2.14 (1.07)	1.43 (1.13)
**EQ-5D-5L utility**^**a**^**(−0.594–1)**	0.55 (0.22)	0.65 (0.14)	0.69 (0.21)	0.15 (0.26)	0.64 (0.13)	0.76 (0.13)	0.79 (0.13)	0.16 (0.16)
**EQ-5D-5L VAS**^**b**^**(0–100)**	68.57 (11.80)	70.71 (14.84)	76.43 (15.74)	7.86 (20.18)	68.57 (11.07)	85.00 (12.91)	85.86 (16.30)	17.29 (21.00)

*TAU* Treatment as usual, *TT* Trial treatment, *SD* Standard deviation, *PEM* Patient evaluation measure, *ORS* Oedema rating scale, *VAS* Visual analogue scale

^a^A higher score (closer to 1) indicates higher quality of life derived health utility

^b^A higher score indicates better health states

**Table 5:** Intention to treat analysis for primary and secondary outcomes at 4 weeks

	Treatment as usual (***n*** = 7) Mean (SD)	Trial treatment (***n*** = 7) Mean (SD)	Adjusted mean difference at 4 weeks unless stated (95% ***CI***)	Linear regression ***p***-value
**Volumeter (ml)**	490.71 (59.47)	476.43 (103.27)	11.99 (−44.74 to 68.72)	0.651
**PEM (0–100)**	46.20 (19.39)	44.90 (14.57)	8.86 (−2.92 to 20.64)	0.126
**ORS (0**−**6)**				
**0**−**2**	3 (43%)	2 (29%)	1.60^a^ (0.16 to 16.23)	0.692^b^
**3**−**6**	4 (57%)	5 (71%)		
**EQ-5D-5L utility (**−**0.594–1)**	0.65 (0.14)	0.76 (0.13)	−0.87 (−0.25 to 0.07)	0.251
**EQ-5D-5L VAS (0–100)**	70.71 (14.84)	85.00 (12.91)	−14.29 (−31.36 to 2.79)	0.093

*SD* Standard deviation, *PEM* Patient evaluation measure, *ORS* Oedema rating scale, *VAS* Visual analogue scale, *CI* Confidence interval

^a^Adjusted (ORS score dichotomised) odds ratio

^b^Logistic regression

**Table 6:** Intention to treat analysis for primary and secondary outcomes at 12 weeks

	Treatment as usual ***n*** = 7 Mean (SD)	Trial treatment ***n*** = 7 Mean (SD)	Adjusted mean difference at 12 weeks unless stated (95% ***CI***)	Linear regression ***p***-value
**Volumeter (ml)**	473.57 (60.60)	460.00 (97.47)	11.21 (−33.42 to 55.83)	0.591
**PEM (0–100)**	38.60 (18.15)	36.31 (16.98)	6.70 (**−**12.99 to 26.38)	0.470
**ORS (0–6)**				
**0–2**	6 (86%)	4 (57%)	4.29^a^ (0.79 to 63.2)	0.288^b^
**3–6**	1 (14%)	3 (43%)		
**EQ-5D-5L utility (−0.594–1)**	0.69 (0.21)	0.79 (0.13)	**−**0.081 (**−**0.30 to 0.13)	0.422
**EQ-5D-5L VAS (0–100)**	76.43 (15.74)	85.86 (16.30)	**−**9.43 (**−**29.02 to 10.16)	0.312

*SD* Standard deviation, *PEM* Patient evaluation measure, *ORS* Oedema rating scale, *VAS* Visual analogue scale, *CI* Confidence interval

^a^Adjusted (ORS score dichotomized) odds ratio

^b^Logistic regression

### Loss to follow-up (LTFU)

A total of 12 participants (46%) were lost to follow-up, with equal attrition in both groups. A comparison of completers versus those LTFU highlighted differences in certain characteristics. Non-completers tended to be male (58% *n* = 7), younger (mean difference 23.2 years), more likely to have sustained trauma (11 trauma LTFUs, 8 trauma patients completed the study), or had a more acute injury (time since injury 12 days less for those LTFU) with fewer comorbidities, i.e. diabetes.

## Discussion

Pilot trials are a small-scale version of a full-scale trial, and therefore not intended to evidence between-group differences, should they exist. Although the data showed an improvement from baseline to 12 weeks in four of the five outcomes measure, including the primary outcome (hand volume) in favour of TT, the high loss to follow-up and wide confidence intervals provide limited information about the treatment effect. The baseline difference between the groups in the time since injury should also be considered, as those in the treatment-as-usual group were 12 days further since their injury, indicating a longer duration of oedema at baseline than those in the TT group, and possibly responding less well to the intervention. A definitive trial should be pragmatic in its inclusion and exclusion criteria; however, there are certain conditions which can develop as a consequence of trauma or surgery that may delay recovery or exacerbate hand oedema meaning participants respond less well to an intervention, such as CRPS. In a definitive trial, a subgroup analysis could be performed, if adequately powered, on patients who enter the trial with suspected or confirmed CRPS or who develop it whilst enrolled in the trial, to compare their results to those of the rest of the group.

### Recruitment and retention

The study failed to recruit to its target of 100 participants due to a lack of suitable patients within the recruitment period. One plausible explanation is that hand oedema was not as prevalent as first thought; however, there were other factors which also contributed to this. Recruiting hand therapists reported that, on occasions, they were too busy to discuss the trial with a participant who was potentially eligible. The PI was not always notified of these, and therefore, the number of patients assessed for eligibility recorded in the CONSORT diagram is likely to be underestimated. Having only one blinded assessor also affected recruitment, as seven eligible patients were unwilling to wait for the assessor to be available in order to recruit them into the study. There were also challenges with coordinating participants flow through the trial pathway as some participants were discharged by the treating therapist prior to the end of 12-week trial and did not return for their planned final study assessment. Due to therapy appointment cancellations and rescheduling being dealt with by an administrative team, who were unaware of patient’s enrollment in the study, the PI was not always informed of changes to participants’ therapy appointment which meant the PI was not aware or not available to complete some of the follow-up assessments. The amount of time, resource and energy required for a busy acute clinical team to manage their caseloads as well as recruit to a trial were underestimated. Adams et al. [28] in their paper examining the barriers and opportunities for enhancing patient recruitment and retention in clinical studies report “tension between clinical and research workloads was seen to interrupt patient recruitment into studies, despite funding arrangements to manage excess treatment costs”. The findings from their study identify a “perceived gap in national provision for dealing with the additional burden that research could place on clinical teams”. Despite the regular presence of the PI in the department and efforts to keep staff engaged and motivated in the trial, there were barriers at an individual, departmental and organisation level, with limited understanding from clinical managers of how best to support this type of project highlighting a potentially systematic issue with conducting research in acute NHS trusts.

### Acceptability and adherence of treatments

During the participant acceptability to treatment interviews, comments were made regarding the appearance of both the glove and kinesiology tape after they had been used for a while (see Additional file 3). One participant reported having to hide her hand in the glove when it got dirty, and a participant in the TT group reported carrying scissors round in order to trim the edges of the tape when they began to fray. This possible inconvenience may have reduced adherence and acceptability in the trial treatment group. Some participants in the trial treatment group questioned the purpose of needing the kinesiology tape along the entire forearm for isolated digit oedema. Hand therapists educated participants on the process of lymphatic drainage and the purpose of the tape’s position; however, reduced face validity may have influenced the lower acceptability scores for the trial treatment. The two patients who experienced adverse effects to the kinesiology tape and switched to the TAU rated their acceptability based on the treatment they had switched to and had more experience of.

Acceptability of treatments to patients is an important factor to consider when designing a trial. Acceptability can be assessed by using scales or inventories, and specific ones exist for certain settings or populations, with most using a Likert scale. Other factors which could be included in an assessment of acceptability are the following: drop-out rates, discontinuation, reason for discontinuation, and withdrawal rates. Without interviewing patients who were lost to follow-up, it is difficult to attribute this to the acceptability of the treatment alone.

Individual adherence to TAU and TT varied greatly in this study. A greater reduction in mean hand volume was observed in the TT group, despite lower adherence rates than the TAU group. Lower adherence rates could reduce the effectiveness of an intervention; however, the results from this study could indicate that the trial treatment has the potential to be a more effective treatment. The lower levels of adherence in the TT group may have related to kinesiology tape being a novel treatment and may indicate that participants required additional support from clinicians throughout the trial.

Interestingly, kinesiology tape was the most adhered to treatment in the TT group, however, compression, which was the second most adhered to treatment, in the treatment-as-usual group had mean adherence levels that were 20% greater than kinesiology tape.

Adherence was patient-reported using paper diaries. Although cheap and simple, they have many limitations as we are not able to confirm when the diary was completed. Retrospective completion relies on recall, whereas prospective completion may result in hopeful inflation of adherence levels. Asking participants to complete a diary may in itself have raised participants’ awareness and therefore increased adherence as was seen in a study by Moseley [29]. An electronic diary or smartphone app could be considered as an alternative method for recording in real time in a definitive trial.

### Loss to follow-up and adverse effects

Two participants in the trial treatment group reported skin rashes that prevented them from continuing with their allocation treatment. Potential adverse effects of using the k-tape, for example the tape pulling on forearm hairs or skin, were discussed with all participants during the screening and consent process. Closer monitoring of treatment fidelity strategies in particular formal assessment of treatment delivery to ensure adherence to the protocol (24-h rest period from the tape before reintroducing it to the skin) in a future trial could mitigate some of the adverse effects and help reduce the number of protocol deviations. There was a high attrition in this study (54%), and whilst all those lost to follow-up were contacted to try to rearrange their assessment, there were no data on their reasons for discontinuing in the study. There was equal loss to follow-up in both groups in the study, which may indicate their withdrawal was not related to the treatment but to other factors. Due to the acute nature of their hand conditions, participants may have perceived their injury to be short term and assumed their data was of little value to the researcher, particularly if the variable of interest, i.e. oedema, had responded to treatment and resolved. Strategies to ensure improve retention, particularly in participants with characteristics associated with higher risk of loss to follow-up [30], would need to be explored for a definitive trial. These could include follow-up assessments in participant’s home, virtual reviews, phone call or text reminders for follow-up assessments.

### Treatment fidelity

Treatment fidelity is an important aspect of therapy trials. It allows greater confidence that the results obtained were due to the effects of the treatment, and not due to other unknown factors associated with its delivery or implementation. The fidelity strategies employed in this study concentrated on the training of treating therapists. Other strategies, such as case vignettes, competency self-assessments before and after training and a formal assessment of treatment delivery by an independent assessor and monitoring therapist skill maintenance throughout the trial could have been used. A challenge of assessing treatment receipt and enactment in the study was the difficulty associated with accurately establishing if the tape, massage and compression had been applied to the participant’s skin to the required pressure/tension, as described in the protocol, as this would require the use of cutaneous pressure sensors. The use of the written instructions issued to each participant served to reiterate the verbal information and demonstration by the hand therapist. None of the participants commented in their adherence diary about being unsure how to apply the treatments. Whilst the therapists’ training and delivery of interventions may have been adequate, we do not know if patients enacted these as per instructions.

## Conclusion

The results of this pilot trial have identified that some modifications are required in order to make a full-scale trial feasible. Recommendations for a future definitive trial should use a multicentred approach in order to reach target sample size, multiple blinded assessors, and utilise research staff to assist with screening patients, a more in-depth and detailed training plan for treating therapist, and a formal and regular assessment of treatment fidelity by an independent assessor. The use of reminder texts or phone-calls to reduce non-attendance rates and greater emphasis on educating participants on the importance of returning for follow-up. Involving managers and staff from an early stage to increase ‘buy-in’ and wider departmental support for conducting a definitive trial in a busy acute clinical department should also be sought.

## Supplementary Information

**Additional file 1.** Participant oedema treatment acceptability questionnaire.

**Additional file 2.** Participant adherence diary.

**Additional file 3.** Comments from participant acceptability interviews.

## Data Availability

The datasets used and/or analysed during the current study are available from the corresponding author on reasonable request.

## Abbreviations

TT: Trial treatment
TAU: Treatment as usual
ITT: Intention to treat
HRA: Health Research Authority
REC: Research ethics committee
TiDier: Template for the intervention description and reporting
CONSORT: Consolidated Standards of Reporting Trials
ORS: Oedema rating scale
PEM: Patient evaluation measure
PROM: Patient-rated outcome measure
VAS: Visual analogue scale
PI: Principal investigator
SD: Standard deviation
CI: Confidence interval
