# Supplementary figures and images for: A pilot single-blind parallel randomised controlled trial comparing kinesiology tape to compression in the management of subacute hand oedema after trauma
Source: Pilot Feasibility Stud. 2022 Mar 26;8:72. doi: 10.1186/s40814-022-01023-1 (PMC8962097; doi:10.1186/s40814-022-01023-1)

**Additional file 1. Participant oedema treatment acceptability questionnaire**


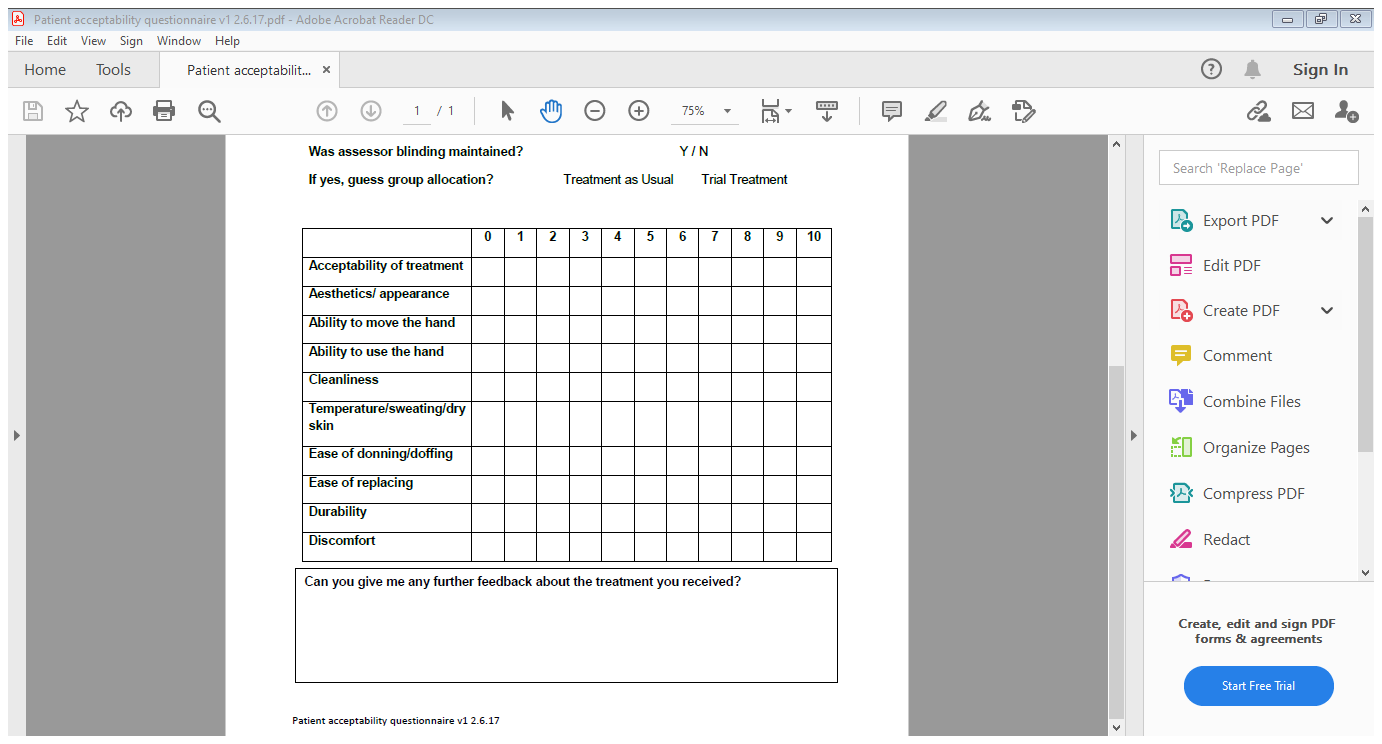

Supplement: Supplementary file 1 — Additional file 1. Participant oedema treatment acceptability questionnaire. [file 40814_2022_1023_MOESM1_ESM.docx]

**Additional file 2. Participant adherence diary**


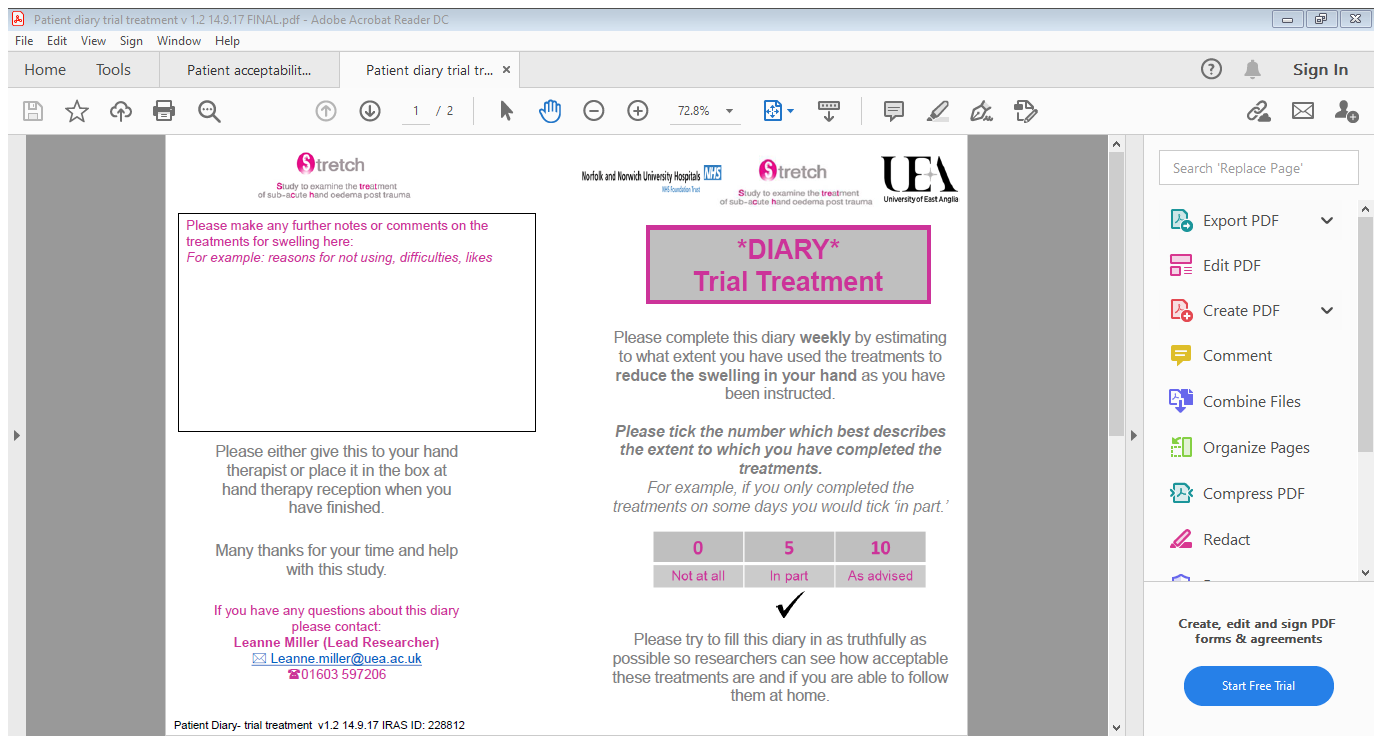


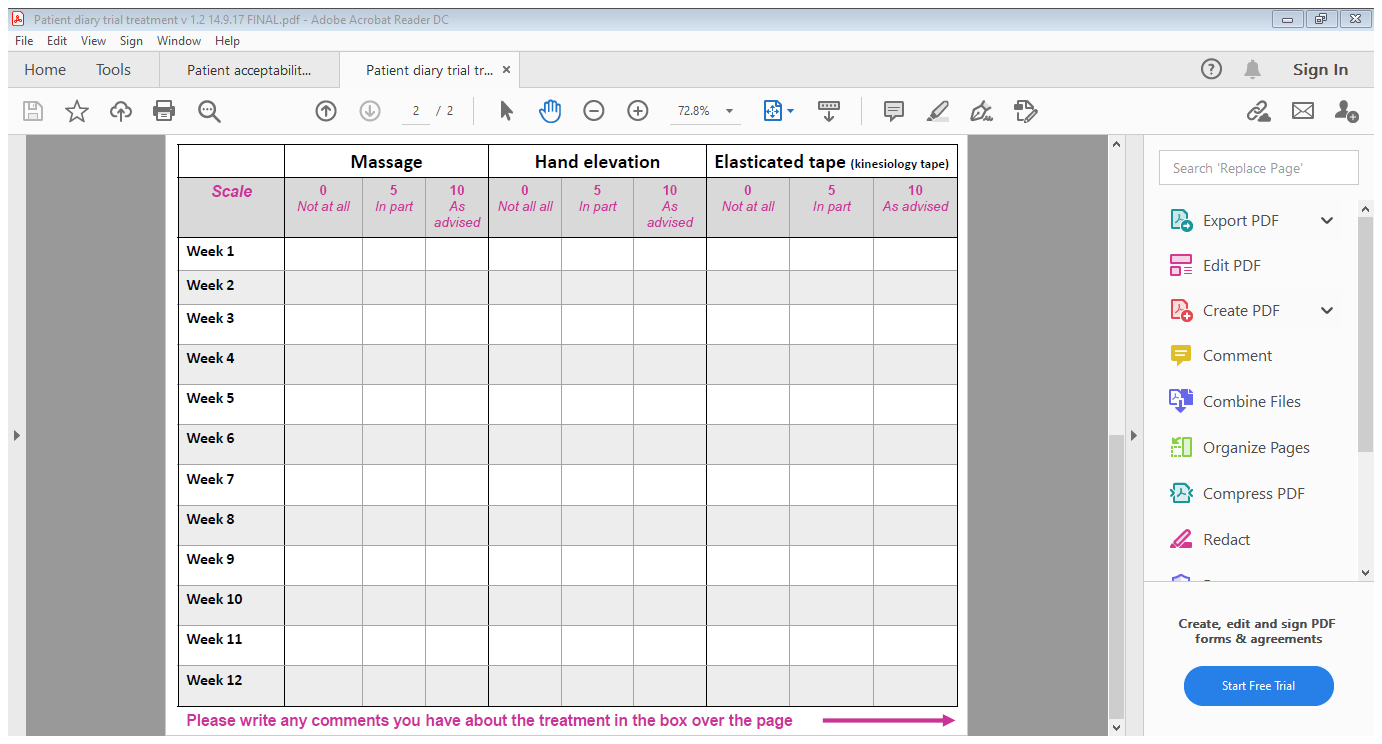

Supplement: Supplementary file 2 — Additional file 2. Participant adherence diary. [file 40814_2022_1023_MOESM2_ESM.docx]
