# Supplementary material for: A pilot single-blind parallel randomised controlled trial comparing kinesiology tape to compression in the management of subacute hand oedema after trauma
Source: Pilot Feasibility Stud. 2022 Mar 26;8:72. doi: 10.1186/s40814-022-01023-1 (PMC8962097; doi:10.1186/s40814-022-01023-1)
Supplement: Supplementary file 3 — Additional file 3. Comments from participant acceptability interviews. [file 40814_2022_1023_MOESM3_ESM.docx]

**Additional file 3. Comments from participant acceptability interviews**

The control treatment group received a diary with ‘compression’ in the final column.

| **Treatment as usual** | **Trial treatment** |
| --- | --- |
| *“It was inconvenient to notice it was dirty as I had to hide my hand”*  *“It was tight at first- I wondered if my fingers were going blue”*  *“[the glove] gives strength to my hand”*  *“Difficulty getting it on but once on was fine”*  *“I preferred it on than off”*  *“Movement was easy when the glove was off” [as a result of wearing the glove]*  *“If I did my arm again- I would want the glove”*  *“Getting glove on was a bit of a job at first”*  *“It helped and assisted hand to do things- when it was off I was a bit more hesitant”*  *“It rubbed slightly in the webspace”*  *“I couldn’t wear it at work as I am a builder”*  *“I washed it regularly but it got dirty very quickly”*  *“I wondered if it was doing anything, felt it needed to be tighter”*  *“[the glove] frayed at the edges”* | *“Looks tatty after a few days”*  *“I needed to keep trimming the edges so I carried scissors round with me”*  *“Didn’t stick on digits” “needed to put fresh {tape} on every day as went loose and stringy at ends”*  *“Tricky to change if dominant hand is injured”*  *“Discomfort when changing the tape as it pulled off my hairs”*  *“I work in the food industry and was unable to wear it at work so I had to replace it daily”*  *“I felt it pulling and squeezing, a feeling of warmth”*  *“It looked untidy at the [finger] tips”*  *“It worked!”* |
